# Copenhagen longitudinal study on quality of life in a young adult population with gliomas

**DOI:** 10.1007/s00701-026-07028-6

**Published:** 2026-09-07

**Authors:** Katrine Arnkjær, Mette Bruun-Pedersen, Esben Meulengracht Flachs, Anne Norup, Jane Skjøth-Rasmussen

**Affiliations:** 1 Department of Neurosurgery, Copenhagen University Hospital, Rigshospitalet, Denmark; 2 Occupational and Environmental Medicine, Copenhagen University Hospital, Bispebjerg Hospital, Copenhagen, Denmark; 3 Neurorehabilitation Research and Knowledge Centre, Copenhagen University Hospital, Rigshospitalet, Denmark; 4 Department of Neuroscience, University of Copenhagen, Copenhagen, Denmark; 5 Department of Clinical Medicine, University of Copenhagen, Copenhagen, Denmark; 6 Danish Comprehensive Cancer Center (DCCC), Network for Adolescents and Young Adults Cancer Research in Denmark (NAYAcare DK), Aarhus, Denmark

**Keywords:** Health related quality of life, AYA (Adolescence and young adults), Glioma, Brain tumor, Neuro-oncology, Patient-reported Outcome

## Abstract

**Background:**

Patients diagnosed with glioma in the brain are prone to lowered quality of life (QoL). The risk of neurological symptoms and emotional burden may restrict participation in everyday life.The glioma diagnosis is considered chronic with a limited overall survival rate, which is particularly concerning for adolescent and young adults (AYA).

**Methods:**

In a longitudinal and prospective study with consecutive inclusion, we followed the QoL in glioma patients aged 18–35 (*n* = 46). Assessment was conducted online with the self-report measures Functional Assessment of Cancer Treatment-Brain (FACT-Br) and Major Depression Inventory (MDI). The assessment was performed consecutively pre- and postoperatively four times per year.

**Results:**

On group level we found that the higher burden of pain, lack of energy and greater burden of seizures and decreased subjective cognitive functioning, significantly predicted the total level of QoL. Tumor grade, time since surgery, sex or number of neurosurgical interventions were not found to significant predict the overall QoL.

**Conclusion:**

The results underline the importance of optimizing the seizure control and treating pain sufficiently as well as assessing fatigue and cognitive functions. By routinely screening the patients with a standardized QoL-questionnaire, unmet needs can be clarified, and rehabilitation be optimized to prevent deterioration in QoL.

**Supplementary Information:**

The online version contains supplementary material available at https://doi.org/10.1007/s00701-026-07028-6.

## Introduction

Being diagnosed with a brain tumor is a devastating experience and might lead to despair and emotional distress for the patient, leading to a higher risk for developing lowered Quality of Life (QoL). The incidence for all types of tumors in the brain and the central nervous system is 25.34 per 100,000 population, where of 22.9% are gliomas [33]. The malignancy is classified in accordance with WHO’s tumor classification: low-grade (grade 1 and 2) and high-grade (grade 3 and 4) [24]. The glioma diagnosis is considered chronic with a limited overall survival rate of 1–15 years (median) depending on the type and grade [22, 39]. Although a lower graded tumor indicates a possible longer survival, more than 70% of the gliomas will transform into a higher grade over a decade [21] Despite the dismal prognosis, patients with lower grade tumors might experience longer periods without progression in their disease.

Ultimately, there is a risk of neurological and functional deterioration over years [19]. Both the disease and side effects from the treatment (brain surgery, radiation- and/or chemotherapy) might influence the emotional and cognitive wellbeing as well as physical abilities [50]. Consequently, this can become a barrier for living a regular everyday life and change the perspectives of the patient’s life [36, 40, 50]. An early assessment and management of rehabilitation needs is therefore recommended [4].

Quality of Life (QoL) is a subjective and multi-dimensional construct [10]. As such, the subjective perception of QoL is of great importance to understand how the individual’s values, internal standards and life-perspectives may change over time during a disease course [35]. Thus, to highlight the patients’ subjective experience, patient-reported outcome data is a preferred research method.

Clinically, QoL is an important subject to discuss during the decision-making regarding treatment options, where individual preferences should be considered to maintain a high level of QoL for as long as possible [8, 9, 14].

The adolescent and young adult population (the AYA population) is a vulnerable patient group with specific needs regarding treatment and rehabilitation [12]. In this age-group cancer is one of the leading causes of death [15] and although the survival of young cancer patients has increased, the mortality rate of young CNS cancer patients has remained stable over the years [12]. The young cancer patients face a variety of issues related to their disease: physical (neurological, neurocognitive, endocrinological, infertility, change in physical appearance) as well as psychosocial and practical problems [42, 45, 50]. These symptoms limit the young patients’ ability to participate in a normal everyday life. The limited participation in social activities affects relationships with peers and/or significant others and difficulties with sexual function and intimate relationships are both frequent issues in the patient group [12]. For the young glioma patients planning to start a family, a potential pregnancy holds the risk of tumor progression [30, 31, 38, 51].

Thus, the symptoms when having a glioma are complex, multifaceted and exhibit a potential risk for the young patients’ well-being and QoL.

To our knowledge no previous studies have investigated longitudinal patterns of QoL in young glioma patients. Longitudinal design is important since the symptom burden from glioma disease evolves over time. Other studies have shown that young patients as a group face distinct challenges linked to their age. Consequently, our study will provide a novel insight into those challenges over time.

We aimed to investigate the longitudinal symptom burden related to QoL and depression in a young glioma patient group. Further, we wanted to identify the prognostic factors for QoL in a young glioma patient group.

## Material and methods

The study was carried out as an online questionnaire-based study with a longitudinal prospective study design. This study offers continuous data on QoL that spans up to 5.5 years with multiple answers for each patient every year. The measure points are spread out in time and not linked to specific treatments or MR-scans or treatment, which could be suspected to have an instant impact on the perception of QoL.

### Participants

**Fig. 1:**
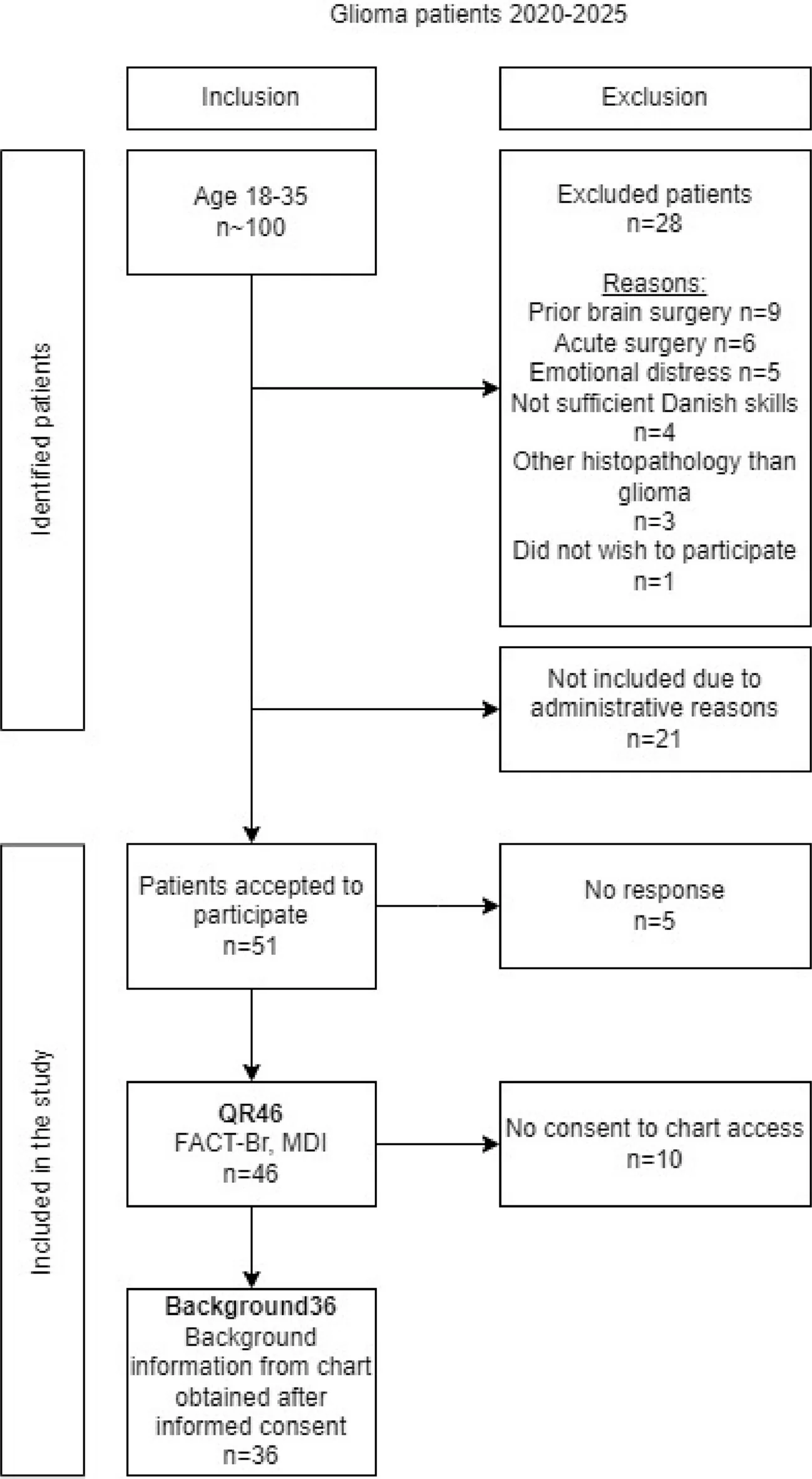
Inclusion of young glioma patients aged 18–35 at Department of Neurosurgery, 2020–2025

Patients aged 18–35 years suspected of having a glioma and planned for a neurosurgical procedure were consecutively included in the study (*n* = 46) constituting the main group subsequently referred to as QR46. Inclusion took place preoperatively during the first consultation at the neurosurgical department at Copenhagen University Hospital – Rigshospitalet (see Fig. 1).

Exclusion criteria included: prior tumor resection, acute or subacute tumor resection before inclusion, other types of tumors than glioma, other severe physical or mental illnesses, not-sufficient Danish skills, severe aphasia or documented severe substance abuse. Patients with severe cognitive impairment and/or acute severe emotional distress after receiving their diagnosis were excluded due to ethical reasons. Individual assessment of cognitive and emotional ability to participate was completed by a trained research nurse. Background information was obtained by chart review after informed consent.

In total 28 patients (28%) were excluded, in accordance with the exclusion criteria or did not wish to participate. A group of patients were not included due to administrative reasons, mainly due to reallocation of the research nurse during the COVID-19 pandemic.

For the main group QR46 only questionnaire-data and age in the span 18–35 years at inclusion was known. For the subgroup referred to as Background36 (*n* = 36) additional data from the patient chart was available as well (mean age 27(SD = 3.8); 50% women). The analysis is divided due to differences in information-level: one analysis regarding each group.

During the first visit to the clinic, the majority of participants from Background36 were employed (64%) and lived with a partner (56%). Most patients were diagnosed with astrocytoma (56%). The most frequent tumor grade was grade 3 (42%). Adding the grade 4 tumors (8%), half of the patients were diagnosed with an HGG (50%). The most frequent tumor location was in the frontal lobes (44%) and a GTCS was the most frequent onset symptom (42%). After the first surgery, most patients (53%) were referred to active observation with pre-scheduled MR-scans and no oncological treatment. Most patients (58%) had no new neurological motor symptoms after the first intervention, and the majority (75%) were discharged directly to home. A rehabilitation plan was made for 36% of the patients. Demographic and clinical background data are summarized in Table 1 respectively Table 2.

**Table 1:** Demographic characteristics at the patients’ first visit

		Percent
*N*	36	
Age in years mean (median) SD	27 (27)3.8	
Sex, *n* (%)		
Male	18	50
Female	18	50
Educational level, *n* (%)		
Low	15	42
Middle	8	22
High	11	31
Unknown	2	6
Job status (%)		
Employed	23	64
Student/apprentice	10	28
Un-employed	3	8
Cohabiting with (%)		
Parents	7	19
Partner	20	56
Roommate(s)	2	6
None	7	19
Have children (%)	9	25
Pregnant at surgery date (%)	1	3

**Table 2:** Clinical background characteristics

Tumor type		
Oligodendroglioma	7	19
Astrocytoma	20	56
DNET	1	3
Glioneural tumor	2	6
Central neurocytoma	1	3
Ependymoma	1	3
Ganglioglioma	1	3
Glioma (NOS)	3	8
Tumor grade (WHO classification)		
Grade 1	2	6
Grade 2	11	31
Grade 3	15	42
Grade 4	3	8
Non-classifiable	5	14
Hemisphere		
Left	16	44
Right	19	53
Midline	1	3
Tumor location		
Frontal	16	44
Temporal	6	17
Parietal	3	8
Occipital	1	3
Multi lobar	8	22
Thalamus	1	3
Other	1	3
Debut symptom(s) described in chart (More symptoms per patient possible)		
GTCS	15	42
Focal seizures	8	22
Tactile symptoms	3	8
Visual disturbances	5	14
Disturbances in consciousness	1	3
Headache	7	19
Dizziness	2	6
Motor symptoms	4	11
INTERVENTION AND TREATMENT		
Neurosurgical intervention		
Stereotactic biopsy	4	11
Resection awake	13	36
Resection asleep	19	53
Number of neurosurgical interventions		
1 intervention	22	61
2 interventions	6	17
3 interventions	2	6
4 interventions	5	14
5 interventions	0	0
6 interventions	1	3
Further treatment		
Oncological treatment	16	44
Active observation	20	56
REHABILITATION NEEDS AS DESCRIBED IN CHART		
Motor symptoms after surgery		
No symptoms	21	58
Symptoms present	15	42
Cognitive symptoms after surgery – as described by neuropsychologist		
Symptoms present	9	25
No symptoms	2	6
Not formally tested post-surgery	25	69
Discharge		
Discharged home	28	78
Discharged to local neurological department to further assessment	7	19
Underwent second surgery before discharge	1	3
Rehabilitation plan		
Discharged with rehabilitation plan	13	36
Discharged without rehabilitation plan	23	64

### Participation

**Fig. 2:**
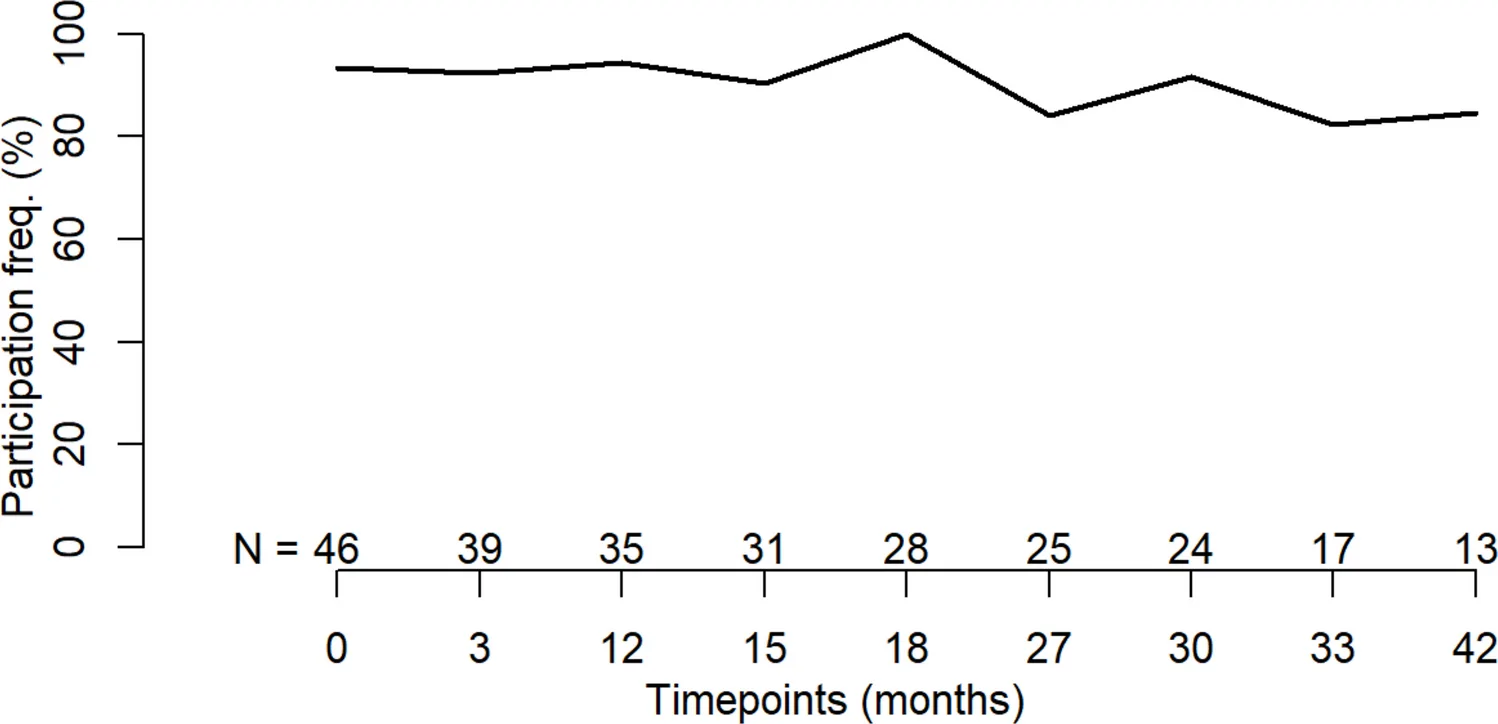
Graph showing the percentage of participation in FACT-Br questionnaires by timepoints (months). *N* = number of patients receiving the FACT-Br

The participation rate for the QR46-group in the FACT-Br varies from 67 to 100% (median: 90; mean: 87) (see Fig. 2) of patients eligible to answer the questionnaires. The difference in number of questionnaires filled out (median: 5; mean: 5.6; SD: 3.7) and the number of patients participating (N) is linked to time. Median participation time (time from first to last answered FACT-Br-questionnaire) is 579 days (SD: 576; mean: 708). After 5.5 years 3 patients have filled out 13 FACT-Br questionnaires, which is explained by the continuous inclusion in the study, consequently not due to drop-out.

## Outcome measures

### Quality of life

#### FACT-Br

The self-report measure Functional Assessment of Cancer Treatment-Brain (FACT-Br, version 4, Danish version) was chosen as outcome measure for QoL. The questionnaire is disease-specific for patients with brain cancer [49] and is an augmentation of the FACT-General (FACT-G), which is the core questionnaire in the FACIT-system [11]. FACT-Br consists of 50 items, scored on a Likert-like scale (0–4) with a total score of 0–200. A high score indicates a higher level of well-being. FACT-Br was chosen since it covers issues related to both low-grade and high-grade tumor patients and does not focus on the side-effects from oncological treatment [25].

A new subjective measure of cognitive abilities introduced by Zarrella et al. (2021) (the Cognitive Index (FACT-Br-CI)), derived from the FACT-Br questionnaire [52] was included in the analysis. It consists of nine items from FACT-Br (see Table 3).

**Table 3:** FACT-Br-Cognitive Index – item number from FACT-Br and description

FACT-Br-Cognitive Index	Item number	Description
	Br1	“I am able to concentrate”
	Br3	“I can remember new things”
	Br8	“I am able to find the right word(s) to say what I mean”
	Br9	“I have trouble expressing my thoughts”
	Br11	“I am able to make decisions and take responsibility”
	Br13	“I am able to put my thoughts together”
	Br15	“I am able to put my thoughts into action”
	Br16	“I am able to read like I used to”
	Br17	“I am able to write like I used to”

### Depression

#### MDI

Depression symptoms and severity were assessed with the Major Depression Inventory (MDI) [2]. The measure was chosen because of its quick administration and validity, when assessing the severity of depression [2, 3, 29]. It consists of 12 items scored on a Likert-like scale from 0 to 5. MDI can be used as a ranking scale to assess severity of depression symptoms by adding up a total score for the 12 items, where a score below 21 indicates no depression, 21–25 points indicates mild depression, 26–30 indicates moderate depression and a score from 31 and above indicates severe depression [2].

## Procedure

The data collection was longitudinal and prospective with a continuous inclusion of patients with a suspected glioma diagnosis. The study period involved consecutive inclusion and a subsequent follow-up over 5.5 years from February 2020 to September 2025. The patients answered a 12-month cycle of questionnaires through a web-link to a database in RedCap. The patients filled out FACT-Br pre-operatively and at 3- and 12-month follow-up. At 6-month follow-up MDI was filled out. If a new neurosurgical event happened the sequence of questionnaires was reset. However, time points were still counted from first neurosurgical event. Otherwise, the cycle of questionnaires was repeated until September 2025 or until the patient decided to withdraw consent. To avoid interference from emotional distress due to hospital visits and MR-C results, the questionnaires were sent out independently of treatments or investigations other than new neurosurgical procedures.

The histopathological diagnosis was confirmed by a neuropathologist. Up until December 2021, the WHO-2016 classification of tumors was used. Consequently, from January 2022 and onwards the WHO-2021 classification of tumors was used.

### Missing data

Missing data in the FACT-Br were handled statistically in accordance with the guidelines from FACIT.org [16] where a missing value is substituted with the average score for the subscale.

### Statistical method

Descriptive statistics for all demographic, background and outcome variables were computed. Means and SD for raw scores were calculated on each of the outcome measures. For each scale variable, development in QoL after resection was analyzed in linear GEE-models allowing for with-in subject correlation. Predictors were included based on existing studies and were included as covariates to study differences in development between groups.

T-tests were used to compare the FACT-G total score among patients to norm values.

### Deference to the participants need of support

The emotional burden of continuously being confronted with one’s disease, as well as the perceived burden of filling out extensive self-report measures, are well known concerns in longitudinal self-report research [1]. Participants were provided with contact information to the research nurse and were encouraged to take contact in between the planned follow-up consultations in case the need for support emerged.

## Results

### Depression symptoms in the QR46-group

Depression symptoms (ICD-10 criteria) in the QR46-group were present in four patients (9%) at 6-month follow-up (see Table 4). The number decreases over time. After 51 months (4.25 years) no patients from the QR46-group fulfilled the ICD-10 criteria for depression on the MDI.

**Table 4:** MDI – depression symptoms in the QR46-group

Time [month]	N. Freq. completed	MDI total score (Mean)	SD	Depression Symptoms MDI (used as diagnostic instrument, ICD-10)			Depression severity MDI (used as rating scale)		
				Mild	Moderate	Severe	Mild	Moderate	severe
*6*	*33*	13.7	10.4	2	1	1	2	2	3
*21*	*27*	13.7	9.8	1	1	1	1	2	3
*36*	*14*	14.0	11.0	0	1	1	1	0	2
*51*	*9*	10.7	6.9	0	0	0	0	1	0

When using the MDI as a rating scale, it shows that seven patients (19%) scored above cut-off at 6 months-follow-up, indicating a symptom burden equaling a depression, although not necessarily fulfilling the ICD-10 criteria. After 66 months (5.5 years) none of the patients in the QR46-group report a burden similar to depression (see Table 4).

### QoL at different timepoints for the QR46-group

Mean scores of FACT-G Total (the core questionnaire in FACT) are between 75.8 (preoperatively) and 92.6 (at 42 months) for the QR46-group (See Table 5). We found a significant development in the FACT-G total over time (*p* = 0.012). The development in FACT-Br was not significant (*p* = 0.07). After 33 months the number of participants (*n* = 14) is too low to draw stable conclusions on (see Table 5).

**Table 5:** QR46: FACT-Br Total and FACT-G Total

Time [month]	N.Freq (Questionnaire completed)	FACT-Br		FACT-G	
		Mean	SD	Mean	SD
0	43	144.9	23.3	75.8	12.4
3	36	158.0	26.2	83.5	16.0
12	33	154.8	27.6	82.1	15.5
15	28	154.6	30.0	82.0	18.5
18	28	153.9	27.6	82.7	16.2
27	21	155.1	27.3	82.8	15.5
30	22	160.1	18.8	86.0	11.7
33	14	163.1	24.1	89.2	14.9
42	11	169.2	17.1	92.6	12.4

### Predictors of QoL-total score in the QR46-group

To assess fatigue, pain and the burden of seizures, we included an analysis on selected items from FACT-Br. Subjective cognitive functioning was assessed with the sub-scale FACT-Br-CI (see Table 3).

To assess development in QoL over time “time since first questionnaire” (pre-operatively) was included. The linear GEE analysis was adjusted for the FACT-subscale “Emotional well-being” (EWB) and for the fact that 3 participants answered their pre-operative questionnaire after the surgery. The analyzed predictors are summarized in Table 6.

**Table 6:** FACT-Br –predictors for QoL in the QR46-group

Predictor	Item/index	Description	Significance (*p)*
Fatigue	GP 1	”I have a lack of energy”	< 0.001*
	GP 7	“I am forced to spend time in bed”	0.14
Pain	GP4	”I have pain”	< 0.001*
Epilepsy burden	Br2	”I have had seizures (convulsions)”	< 0.001*
Subjective cognitive functioning	FACT-Br-Cognitive index	See Table 3	< 0.001*
Time	FACT-Br	Time since first questionnaire	0.78

**Fig. 3:**
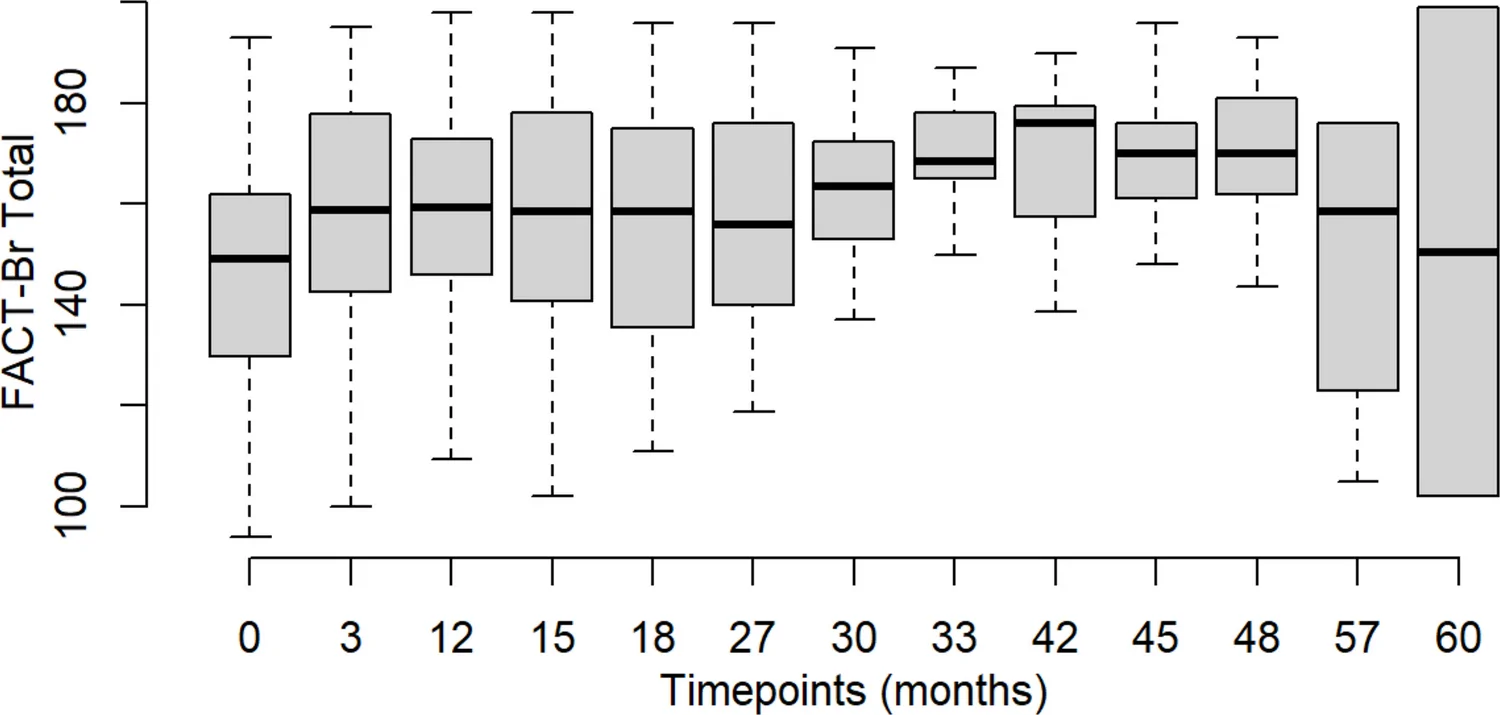
Boxplot showing the development of FACT-Br total score for the QR46-group over time. Grey boxes show 75. percentile and lines indicate 95. percentile of data

For the QR-46-group we found that higher burden of pain (*p* < 0.001), lack of energy (*p* < 0.001),greater burden of seizures (*p* < 0.001), and a low FACT-Br-CI (p < 0.001) predicted the total level of QoL. Each point higher score on item GP4 (*“I have pain”),* gave a reduction of 4.7 (95%-CI: 3.3;6.1), while for GP1 (“*I have a lack of energy”)* the result was a reduction of 4.6 (3.0;6.3). Each point higher score on FACT-Br-CI gave an increase of the total FACT-Br score of 1.8 (1.5;2.1). “Time since first questionnaire” (*p* = 0.78) did not affect the prediction of FACT-Br total score (see Fig. 3). The need to stay in bed (*p* = 0.14) did not affect the prediction of the FACT-Br total score.

For the patients with a classifiable tumor (*n* = 31) the analysis did not find tumor grade (*p* = 0.54), time (*p* = 0.36), sex (*p* = 0.95) or number of neurosurgical interventions (*p* = 0.07) predictive for the FACT-Br total score.

### Development over time in the QR46-group in the subscales in FACT-Br

To assess whether there was a development over time in the FACT-Br subscales in the QR46-group a linear GEE analysis was conducted (see Table 7).

**Table 7:** Development in subscales (FACT-Br) in the QR46-group

Subscale	Significance (*p)*
Physical Well-being	0.092
Social Well-being	0.65
Emotional Well-being	0.003*
Functional Well-being	0.009*
Additional concerns	0.34

We found a significant development over time in the two subscales “Emotional Well-being” (EWB) (0.987 points increase pr. year; *p* = 0.003) and “Functional Well-being” (FWB) (1.085 points increase pr. year; *p* = 0.009) (see Table 7).

The development in the remaining subscales “Social Well-being” (*p* = 0.65), “Physical Well-being” (*p* = 0.092) and the brain specific “Additional Concerns” (*p* = 0.34) was not significant.

### QoL in the background36-group compared to norm group

**Fig. 4:**
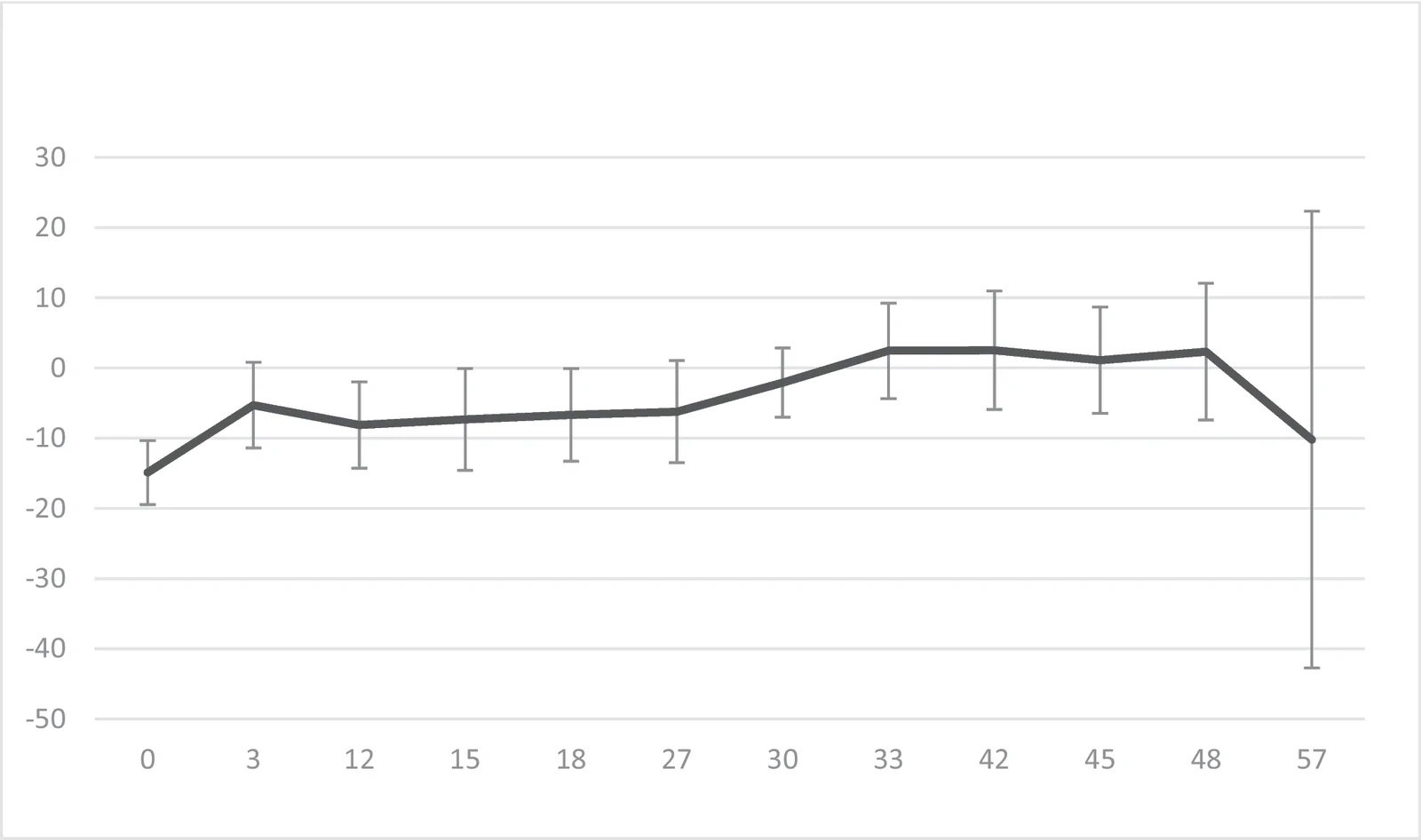
The curve indicates the average difference of FACT-G mean scores in the Background36 to norm scores of young adults by Holzner et al. (2009) by timepoint (months)

A series of t-tests (one at each questionnaire timepoint for the Background36-group with known age) shows that our patients’ mean score on FACT-G was significantly lower (*p* < 0.001) than the norm group by Holzner et al. (2009) (Fig. 4), rising to a level similar to the norm group after 18 months. Since no SD was reported on the norm values in Holzner et al. (2009) it was used as a constant which underestimates the uncertainty resulting in too narrow confidence intervals. The timepoint 60 months is not shown in Fig. 4 due to readability of the graph (see Fig. 4).

Norm values in the background population were collected with the core QoL-instrument FACT-G by Holzner et al. (2009) – omitting the disease specific part (Br) since the questions are not suitable for non-patients [20].

## Discussion

Our analysis shows that our patients in general display lower levels of QoL compared with healthy peers at least during the first 18 months (1,5 years) after first neurosurgical intervention. On a group level, no significant development was found in the overall QoL (FACT-Br). A significant positive development was found in two subscales measuring emotional and functional well-being. Higher burden of pain, lack of energy, greater burden of seizures and lowered subjective cognitive functioning were significant predictors for lowered QoL (FACT-Br).

### Depression

Almost a fifth of our patients reported a symptom burden similar to depression at 6 months-follow up which is thought to be at least a part of the explanation for the lowered QoL in our group. Depression is a frequent and persistent symptom in glioma patients [6, 7, 37] and has been found to be strongly related to overall QoL [27, 34].

### Time as predictor for QoL

We found a relatively stable overall QoL over time in our patient group. Other studies [5, 7, 37, 43] have reported a similar relative stable QoL over time in the low-grade glioma population. Reports on QoL over time, however, are in general burdened by a heterogeneity in study design, measure points, QoL-instruments used and lack of use of normative group and cut-off values [13, 17, 37, 43] making direct comparisons difficult.

The stability in QoL over time is, as such, not a positive finding since the baseline scores for the glioma patients tend to be low [37] which was also found in our patient group when compared to a young healthy norm group [20]. Although no development was found in FACT-Br, there was a significant development in the core questionnaire FACT-G over time. This might suggest that there is an improvement of general QoL over time but not reaching a significant level when the neurological symptoms are included (the disease specific part of the questionnaire).

The stable overall QoL in our patient group may be due to the short time from disease onset since severe symptoms are expected later in the disease course. The longest participating patients in our study had their first brain surgery 5.5 years prior to data retrieval, and the mean participation time was 1.9 years (708 days; SD = 576). At least for the LGG patients there might be longer stable periods with no progression in their illness and no active treatment – although diffuse symptoms like fatigue, pain as well as depression might still be present in those periods [5]. The classification and prognosis for the patients have become more precise, due to an increasing knowledge on the molecular background of CNS tumors. This has led to a more positive trajectory for the grade 3 glioma patients, that likely experience longer stable periods similar to the LGG patients [4]. Since it is well-known that oncological treatment may lead to undesirable side-effects, both cognitively and physically, a change in QoL might be expected even for the initial LGG-patients as the disease evolves and the treatment changes [6, 26]. It may also be speculated that some adaptation to the new life with a chronic disease happens over time and with several follow-up scans showing stable disease.

### Fatigue

We found that higher levels of fatigue significantly predicted a lower overall QoL. This association has been found in glioma patients in several studies supporting our finding [6, 7, 34, 37, 43, 46].

Fatigue is a persistent and frequent symptom after neurological injury or disease with a prevalence rate ranging from 39 to 77% in LGG patients [46]. Fatigue is a well-known factor influencing the patients’ ability to participate in their everyday-life and is as a clinical construct thought to be trans diagnostical [28, 48]. In other studies fatigue has been found to be a key bridge symptom in the AYA brain tumor patients, meaning that difficulties for the patient seem to spread from that symptom [41]. Beside the neurological impact from the tumor and surgery, side-effects from anti-epileptic drugs, pain relieving medicine and oncological treatment might contribute to the experienced fatigue in active treatment periods [46] which to some degree might explain the fatigue symptom burden in our patient group.

### Epilepsy burden

We found that a greater epilepsy burden was associated with lowered over all QoL. This finding is supported in a review on QoL in glioma patients [37] where epilepsy burden is the most frequent negatively influencing factor across the reviewed studies.

### Cognitive functions

We found that lower subjective cognitive functioning was associated with poorer overall QoL scores. This observed association is in accordance with the findings from other studies [43] whereas others have found the same trend but without reaching a significant prediction [23].

Trans-diagnostically, the association between objective and subjective cognitive functioning is inconsistent. Objective and subjective cognitive functioning are largely not overlapping constructs and are suggested to reflect different aspects of cognitive functioning that complement each other [32, 47] which is of big importance when comparing studies on cognition in the glioma literature. Cognitive deficits found in objective performance based cognitive testing are frequent and persistent in the glioma patients [1, 6, 37] and might be present and prevalent at least 5 years after diagnosis [6].

Forty-three percent of our patients received oncological treatment after first surgery. Since radiation therapy is associated with lower cognitive performance and lower QoL outcome [6] this might to some degree explain the cognitive deficits our patients experience. We did not find that the number of surgical interventions and tumor grade affected the overall level of cognitive functioning in our patient group.

### Clinical impact

The results from the project improve knowledge of young glioma patients and their specific needs along their disease trajectory. This knowledge could improve guidelines for rehabilitation and general support for the young glioma patients by pointing out the prognostic factors regarding QoL and by obtaining experience with monitoring QoL continuously in this patient group. The need for support and interventions could be met earlier if clinicians early in the treatment course were able to identify patients at risk and thereby preventing lowered QoL and emotional distress for the patient.

The significant predictors we found for overall QoL (burden of seizures, pain, fatigue and decline in cognitive functioning) are all factors that can be treated or at least tried to be minimized. This suggests that clinical priority on those factors can minimize the distress followed by having a glioma on the patients’ QoL.

The high participation rate suggests that a systematically assessment of QoL can be implemented in the clinic without much extra work needed and thereby enhance relevance of topics covered at follow-ups in the clinic and enhance the overall treatment outcome for the patient.

## Compliance and future adaptations

In the present setup the FACT-Br and MDI questionnaires are not sent out at the same time. This was chosen to minimize the burden for the patients and to reduce drop-out. Based on the high participation rate, FACT-Br and MDI will in the future be sent out simultaneously, thus allowing additional analyses on the relation between depression and QoL.

In the future, we plan to include an analysis on the development of individual QoL level as suggested by Habets et al. [18]. An analysis on the association between QoL and patients’ socio-economic situation and oncological treatment will be included as well as neuropsychological testing as objective measure of cognition. The semi-objective Karnofsky Performance Status (KPS) will be applied as a broad measure of functional level making an analysis on QoL and daily function possible.

A qualitative analysis will be conducted to uncover the patients view on rehabilitation outcome and possibly reveal other topics related to QoL than already uncovered with the FACT-Br.

## Strengths and limitations

Strengths include having studied a small under-investigated patient population– the young glioma patients – and followed them over a longer period as their life perspectives are changed due to a new and life-shortening disease. We have monitored their QoL in fixed intervals and these data has contributed to new knowledge in the field of QoL in young glioma patients. The flexible online participation in the study seems to suit the patient group and a high participation rate was seen.

Study limitations include being a single-center study with a small sample size due to the low incidence of glioma combined with the focused age span. The combination of a small sample size and a study-design with consecutive inclusion makes it difficult to generalize and draw firm conclusions on the questionnaire data after timepoint 33 months. As more patients are consecutively included, the questionnaire data will continue to be more complete for future analysis. Neurological symptoms were obtained from the medical chart and were not assessed on a rating scale specifically for the study, which might have led to a more uneven assessment. The present study does not include an analysis of the relationship between socio-economic status and QoL that could have contributed to a broader description of predictors for QoL. The use of online questionnaires holds a risk for other people around the patient to influence the patients’ answers, making the responses less reliable.

Severely emotionally distressed patients who were deemed too burdened to participate were not included in the study due to ethical reasons, which might have contributed to a selection bias limiting the generalization of the results from our study. We had knowledge of the initial oncological treatment after first surgery and discharge from the department of neurosurgery, but no knowledge about further treatment or other factors such as social events that could be thought to influence the general QoL for the patient.

## Conclusion

In this longitudinal study on young glioma patients, we found that a higher burden of pain, lack of energy, greater burden of seizures and low subjective cognitive functioning significantly predicted a lower overall QoL. The young patients’ QoL was stable over time although the QoL-score was significant below the norm-score for healthy young adults for the first year and a half after the glioma diagnosis.

Our results underline the importance of optimizing the young glioma patients’ seizure control and being aware of treating pain sufficiently. Fatigue and impaired cognitive functions are easily overlooked in the clinic as they often need systematically assessment to be detected. By routinely screening the patients with a standardized QoL-questionnaire, that we proved to be feasible over a longer time period, unmet needs can be clarified, and assessment and rehabilitation be optimized.

### Future directions

We encourage clinicians working with this vulnerable and small group of young glioma patients to use this information for additional guidance and further studies. Although the generalizability is limited, our results could however be a platform for future studies elaborating the QoL for this patient group.

## Supplementary Information

Below is the link to the electronic supplementary material.

ESM 1 Supplementary Material 1 (DOCX 14.4 KB)

## Data Availability

All data supporting the findings of this study are available within the paper and its Supplementary Information.

## Abbreviations

AYA: Adolescence and Young Adults
FACT-Br: Functional Assessment of Cancer Treatment—Brain
FACT-G: Functional Assessment of Cancer Treatment—General
GTCS: Generalized Tonic–Clonic Seizure
HGG: High Grade Glioma
LGG: Low Grade Glioma
MDI: Major Depression Inventory
PRO: Patient-reported Outcome
QoL: Quality of Life
WHO-5: WHO-5 Well-Being Index
