# Supplementary material for: Copenhagen longitudinal study on quality of life in a young adult population with gliomas
Source: Acta Neurochir (Wien). 2026 Sep 7;168(1):242. doi: 10.1007/s00701-026-07028-6 (PMC13683255; doi:10.1007/s00701-026-07028-6)
Supplement: Supplementary file 1 [file 701_2026_7028_MOESM1_ESM.docx]

| Supplementary material, table 1: Procedure and order of questionnaires | | | | | |
| --- | --- | --- | --- | --- | --- |
| Round | 1 | 2 | 3 | 4 | 3-month break until repetion of the four rounds |
|  | Preoperatively  (time=0) | 3 months | 6 months | 12 months |  |
| FACT-BR | X | X |  | X |  |
| MDI |  |  | X |  |  |
